# Supplementary material for: Emotional associative memory is disrupted by directed forgetting
Source: Commun Psychol. 2023 Oct 10;1:24. doi: 10.1038/s44271-023-00024-x (PMC11332221; doi:10.1038/s44271-023-00024-x)
Supplement: Supplementary file 1 — Supplementary Information [file 44271_2023_24_MOESM1_ESM.pdf]

Supplementary Information

Emotional associative memory is disrupted by directed forgetting

Anastasia Chalkia*, Niels Vanhasbroeck, Lukas Van Oudenhove, Merel Kindt, Tom Beckers

*Address for correspondence: Anastasia Chalkia

Tiensestraat 102 – box 3712

3000 Leuven, Belgium

Tel +32 16 32 92 08

E-mail anastasia.chalkia@kuleuven.be

Supplementary Methods

Conditioned stimuli were selected from the Snodgrass & Vanderwart (1980) standardized set of images. Practice items included images of a saw, mask, robot, and skateboard. Acquisition items included images of a swing, car, foot, book, guitar, leaf, eye, fork, shoe, clown, cross, bicycle, tent, apple, airplane, tree, box, hat, chair, pipe, scissors, egg, arrow, and house. Novel items presented during the recognition task included images of a key, telephone, candle, balloon, mirror, umbrella, bottle, glasses, bucket, ring, flag, and sun.

Supplementary Results

US selection

As reported in the main text, participants were allowed to select their own US intensity prior to the start of the experiments. In Experiment 1, the average selected US intensity was 6.35mA, while in Experiment 2, it was 6.99mA when all 68 participants were included, and 7.02mA when participants were excluded for reasons of SCR non-responding, thus yielding the final sample of $N = 45$.

Development of SCR across blocks

Besides our preregistered analyses for Experiments 1 and 2, which only included SCR responding during Blocks 2 and 3 of our conditioning procedure, we also examined the development of SCR across all three blocks of the experiments and how it was influenced by forgetting instructions (see Fig. S1). To do so, we conducted additional rm-ANOVAs with Stimulus (CS+, CS-), Instruction (Remember, Forget), and Block (1, 2, 3) as within-participants factors.

In Experiment 1, we observed statistically significant main effects of stimulus ($F(1, 39) = 28.89$, $p < .001$, $\eta_p^2 = 0.43$, 95% CI [0.19, 0.59]) and instruction ($F(1, 39) = 6.50$, $p = .015$, $\eta_p^2 = 0.14$, 95% CI [0.01, 0.34]), as well as a stimulus by instruction interaction (F(1,

39) = 6.95, p = .012, η_p^2 = 0.15, 95% CI [0.01, 0.35]), and a stimulus by block interaction (F(2, 78) = 3.94, p = .023, η_p^2 = 0.09, 95% CI [0.001, 0.21]). In addition, we found a three-way interaction between stimulus, instruction, and block (F(2, 78) = 7.26, p = .001, η_p^2 = 0.16, 95% CI [0.03, 0.29]), suggesting that differential SCR responding evolved across blocks and that the course of this was affected by instruction. In follow-up to the three-way interaction, we conducted paired t-tests to examine the direction of these effects. In Block 1, there was no evidence of a statistically significant difference between SCRs for CS+ items (t(39) < 1), but the difference between CS-R and CS-F did reach significance (t(39) = 3.07, p = .004, d = 0.49, 95% CI [0.15, 0.81]) most likely due to chance, as in Block 1 participants saw all items for the first time, hence, no learning had occurred yet. In Block 2, the effect of the forgetting instructions became evident as SCR was significantly higher for CS+R than for CS+F items (t(39) = 2.90, p = .006, d = 0.46, 95% CI [0.13, 0.78]). No statistically significant differences were observed between CS- items (t(39) < 1), as SCR is not likely to develop in response to safe associations, and thus, it is expected to remain generally low for all CS- items. The same pattern of results was maintained in Block 3, with larger SCRs for CS+R items in comparison to CS+F items (t(39) = 2.47, p = .018, d = 0.39, 95% CI [0.07, 0.71]), but no evidence of significant differences between CS- items (t(39) = -1.53, p = .13, d = -0.24, 95% CI [-0.56, 0.07]). These results indicate that after learning occurs (i.e., after Block 1), forgetting instructions can reliably interfere with the physiological expression of emotional associative memory, reflected here as diminished SCR to CS+F items.

In Experiment 2, we similarly obtained statistically significant main effects of stimulus (F(1, 44) = 55.26, p < .001, η_p^2 = 0.56, 95% CI [0.34, 0.68]) and instruction (F(1, 44) = 7.38, p = .009, η_p^2 = 0.14, 95% CI [0.01, 0.33]), in addition to the significant interactions between stimulus and instruction (F(1, 44) = 8.41, p = .006, η_p^2 = 0.16, 95% CI [0.01, 0.35]), and stimulus by block (F(2, 88) = 6.04, p = .003, η_p^2 = 0.12, 95% CI [0.02,

0.24]). Yet in this experiment, we also observed a main effect of block ($F(2, 88) = 9.94$, $p < .001$, $\eta_p^2 = 0.18$, 95% CI [0.05, 0.32]) and a block by instruction interaction ($F(2, 88) = 5.76$, $p = .004$, $\eta_p^2 = 0.12$, 95% CI [0.01, 0.24]), while failing to obtain a statistically significant three-way interaction between stimulus, instruction, and block ($F(2, 88) = 1.72$, $p = .18$, $\eta_p^2 = 0.04$, 95% CI [0.00, 0.13]). These results suggest that our forgetting instructions had a differential effect across blocks that was not influenced by the stimulus per se. To facilitate comparison between our two experiments, we followed-up these supplementary analyses with paired *t*-tests in each block, just as in Experiment 1. In Block 1, we found no evidence for a statistically significant difference on SCR responding for either CS+ ($t(44) = 2.00$, $p = .051$, $d = 0.30$, 95% CI [-0.002, 0.60]) or CS- ($t(44) < 1$) items, as was to be expected given the lack of learning at the onset of the experiment. In Block 2, and just like Experiment 1, the development of SCR was hindered for CS+F items, as we found a significant difference between remember and forget CS+ items ($t(44) = 4.13$, $p < .001$, $d = 0.62$, 95% CI [0.29, 0.93]). Once more, our instructions did not have a statistically significant influence CS- items in Block 2 ($t(44) < 1$) or Block 3 ($t(44) = -1.22$, $p = .23$, $d = -0.18$, 95% CI [-0.48, 0.11]). Interestingly, even though the pattern of CS+ SCR responding in Block 3 appears very similar between experiments (see Fig. S1), the difference between CS+R and CS+F did not reach statistical significance in Block 3 of the current experiment ($t(44) = 1.15$, $p = .26$, $d = 0.17$, 95% CI [-0.12, 0.47]), perhaps due to differences in standard deviation. In this second experiment, the development of SCR was also swayed by DF instructions, however, we maintain caution in this interpretation given the non-significant statistical findings in Block 3.

Fig. S1. Skin conductance responses across blocks.

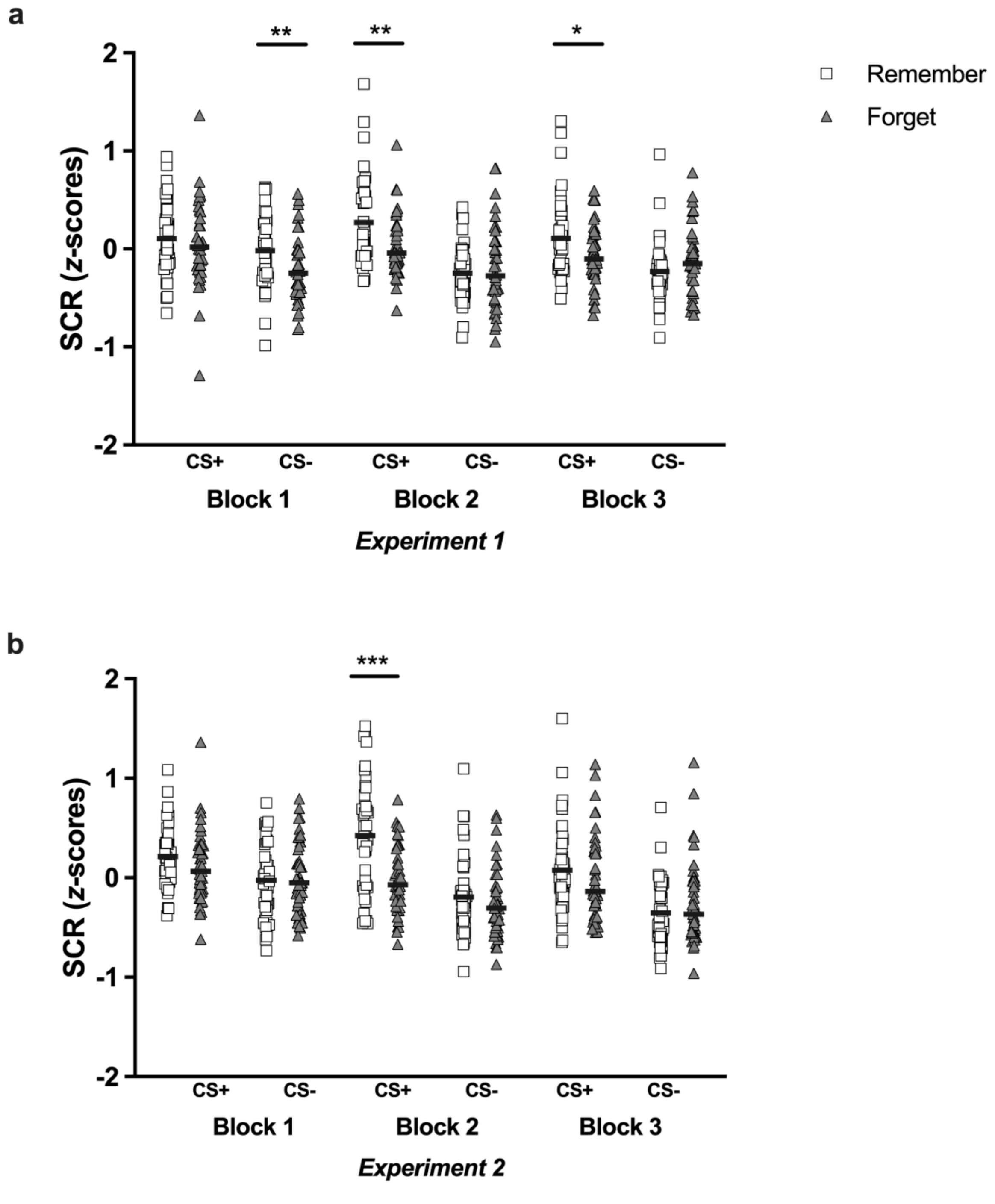

Average SCR per stimulus category, across all blocks, for **a)** Experiment 1, and **b)** Experiment 2. White squares represent Remember items and grey triangles represent Forget items. Solid black lines depict the group means. **p* < .05, ***p* < .01, ****p* < .001

Experiment 2 - Individual differences analyses

In Experiment 2, we also tested whether differences in working memory capacity and certain personality characteristics could differentially influence the DF effect. Descriptive statistics for our individual difference measures can be found in Table S1. Our preregistered correlation analyses using the differential indices calculated for free recall, recognition, and SCR (see Methods) yielded mostly non-significant results and will not be described further. Only one of the comparisons produced a significant finding, showing that participants who scored higher on the BFI-Neuroticism scale had a larger RT difference between congruent and incongruent trials on the Flanker task ($r(43) = .34$, $p = .024$, 95% CI [0.05, 0.57]). Given the lack of any other statistically significant correlations to corroborate this finding, it should be interpreted with caution. Thus, our correlation analyses did not allow us to draw any conclusions about possible individual differences influencing the magnitude of the DF effect for emotional associative memories.

Table S1.
Descriptive statistics for individual difference measures

	Mean	***SD***
FLANKER ACC (%) Congruent	98.32	2.02
FLANKER ACC (%) Incongruent	96.71	3.00
FLANKER RT (*s*) Congruent	0.51	0.08
FLANKER RT (*s*) Incongruent	0.54	0.08
FLANKER RT (*s*) Difference	0.03	0.03
Nback ACC (%)	96.80	2.91
Nback RT (*s*) Hits	0.59	0.09
Nback RT (*s*) False Alarms	0.78	0.23
Nback Hit Rate (H)	0.95	0.05
Nback FA Rate (F)	0.02	0.03
Nback D-Prime	4.01	0.90
Perfectionism	23.31	4.88
Neuroticism	20.62	4.64
Mind-wandering	18.98	3.91

Note. Accuracy (ACC), reaction time (RT)

Experiment 2 - Analyses following SCR exclusions (*N* = 45)

Prior to commencing Experiment 2, we had preregistered an SCR exclusion criterion which eventually led to the exclusion of 21 participants. However, in the main text, we reported analyses for Experiment 2 using a total sample size of 68 for most of our outcome measures (except for SCR), as we had obtained complete datasets for these participants and did not want to disregard these data. To investigate any possible discrepancies between these samples, we repeated all the analyses using a sample size of 45. The findings were almost identical, and rather than reiterating all the results here, we will only briefly report and discuss the few differences detected between the two samples. While item free recall was alike in both samples, when categorizing those items as CS+ or CS-, the sample of 45 participants only showed a statistically significant difference in categorization accuracy between remember and forget items for CS+ trials ($t(44) = 6.98$, $p < .001$, $d = 1.48$, 95% CI [0.77, 2.19]), but not for CS- trials ($t(44) = 2.52$, $p = .083$, $d = 0.54$, 95% CI [-0.05, 1.12]). The CS- comparison did reach significance in the sample of 68 participants, perhaps due to the larger sample size yielding higher statistical power. Performance on the liking ratings was also comparable between samples, except for the follow-up comparison between CS+R and CS+F which was non-significant using a sample of 45 ($t(44) = -2.72$, $p = .15$, $d = -0.40$, 95% CI [-0.88, 0.08]), in contrast to what is reported in the main text with the full sample. Other than that, there were no further differences between the sample including all participants (*N* = 68) and the sample with participants excluded for reasons of SCR non-responding [and technical malfunctions] (*N* = 45).
