# Supplementary material for: Emotional associative memory is disrupted by directed forgetting
Source: Commun Psychol. 2023 Oct 10;1:24. doi: 10.1038/s44271-023-00024-x (PMC11332221; doi:10.1038/s44271-023-00024-x)
Supplement: Supplementary file 3 — Reporting Summary [file 44271_2023_24_MOESM3_ESM.pdf]

nature portfolio

Corresponding author(s): Anastasia Chalkia

Last updated by author(s): Aug 17, 2023

Reporting Summary

Nature Portfolio wishes to improve the reproducibility of the work that we publish. This form provides structure for consistency and transparency in reporting. For further information on Nature Portfolio policies, see our Editorial Policies and the Editorial Policy Checklist.

Statistics

For all statistical analyses, confirm that the following items are present in the figure legend, table legend, main text, or Methods section.

n/a	Confirmed	
☐	☒	The exact sample size (*n*) for each experimental group/condition, given as a discrete number and unit of measurement
☐	☒	A statement on whether measurements were taken from distinct samples or whether the same sample was measured repeatedly
☐	☒	The statistical test(s) used AND whether they are one- or two-sided<br>*Only common tests should be described solely by name; describe more complex techniques in the Methods section.*
☒	☐	A description of all covariates tested
☐	☒	A description of any assumptions or corrections, such as tests of normality and adjustment for multiple comparisons
☐	☒	A full description of the statistical parameters including central tendency (e.g. means) or other basic estimates (e.g. regression coefficient) AND variation (e.g. standard deviation) or associated estimates of uncertainty (e.g. confidence intervals)
☐	☒	For null hypothesis testing, the test statistic (e.g. *F*, *t*, *r*) with confidence intervals, effect sizes, degrees of freedom and *P* value noted<br>*Give P values as exact values whenever suitable.*
☒	☐	For Bayesian analysis, information on the choice of priors and Markov chain Monte Carlo settings
☒	☐	For hierarchical and complex designs, identification of the appropriate level for tests and full reporting of outcomes
☐	☒	Estimates of effect sizes (e.g. Cohen's *d*, Pearson's *r*), indicating how they were calculated

Our web collection on statistics for biologists contains articles on many of the points above.

Software and code

Policy information about availability of computer code

Data collection	Affect 4.0 was used for data collection, a custom-made, freely available software package for behavioral experiments that can simultaneously control stimulus presentation as well as collect data (computer inputed responses and psychophysiological data).
Data analysis	All analyses were performed using JASP version 0.17.1, a freely available statistical package.

For manuscripts utilizing custom algorithms or software that are central to the research but not yet described in published literature, software must be made available to editors and reviewers. We strongly encourage code deposition in a community repository (e.g. GitHub). See the Nature Portfolio guidelines for submitting code & software for further information.

Data

Policy information about availability of data

All manuscripts must include a data availability statement. This statement should provide the following information, where applicable:

- Accession codes, unique identifiers, or web links for publicly available datasets
- A description of any restrictions on data availability
- For clinical datasets or third party data, please ensure that the statement adheres to our policy

The experimental tasks, final datasets generated and used for analyses, and analyses outputs are publicly available on the Open Science Framework (OSF) at https://osf.io/65x7a/.

Research involving human participants, their data, or biological material

Policy information about studies with human participants or human data. See also policy information about sex, gender (identity/presentation), and sexual orientation and race, ethnicity and racism.

Reporting on sex and gender	Sex was determined based on self-report and collected only for demographic purposes. The number of male/female participants is indicated in the manuscript. There were no further analyses looking at sex/gender.
Reporting on race, ethnicity, or other socially relevant groupings	N/A
Population characteristics	See below.
Recruitment	Participants were recruited through the KU Leuven research pool using the SONA systems platform. This is an online platform where students and members from the community can register to take part in experimental research. It could be that certain people would be more likely to register in these platforms than others (i.e., psychology students, people of low income), creating a self-selection bias, but these recruitment procedures are common in experimental research with humans throughout the world.
Ethics oversight	Social and Societal Ethics Committee (SMEC) of KU Leuven

Note that full information on the approval of the study protocol must also be provided in the manuscript.

Field-specific reporting

Please select the one below that is the best fit for your research. If you are not sure, read the appropriate sections before making your selection.

☐ Life sciences ☒ Behavioural & social sciences ☐ Ecological, evolutionary & environmental sciences

For a reference copy of the document with all sections, see nature.com/documents/nr-reporting-summary-flat.pdf

Behavioural & social sciences study design

All studies must disclose on these points even when the disclosure is negative.

Study description	Quantitative experimental within-subjects design
Research sample	Mostly psychology students from KU Leuven, along with members from the community recruited through the university's research pool. This sample was selected for practical reasons, due to their availability to take part in experimental research. Experiment 1: 40 participants (31 female participants), aged between 18 and 39 years (M = 20.85, SD = 4.55). Experiment 2: 68 participants (48 female participants) total, aged between 18 and 42 years (M = 21.44, SD = 4.04); 45 participants (28 female participants) after exclusions, aged between 18 and 27 years (M = 20.82, SD = 2.70). These samples are representative of university research participants.
Sampling strategy	To establish the necessary sample needed for Experiment 1, we ran a small pilot study with 10 subjects (not reported in the manuscript) that yielded very large effect sizes (>1 for all outcome measures). When using these effect sizes in a power analysis, suggested sample sizes were very small (N < 12); to counter undue influence of the data of individual subjects on the results, we preregistered a total sample size of 40. To determine our sample size for Experiment 2, we conducted a power analysis using the effect size we obtained in our critical outcome of interest in Experiment 1 (based on a paired t-test comparison between SCRs for CS +R and CS+F). Setting alpha at .05 and employing said effect size of d = 0.50, a sample of N = 45 in Experiment 2 should have yielded a power of 0.95 to detect this effect.
Data collection	Stimulus presentation (on the computer) during the experiments was done using Affect 4.0, a custom-made, freely available software package for behavioral experiments. Skin conductance was recorded using an isolated skin conductance coupler (LabLinc v71-23, Coulbourn Instruments, Allentown, PA) and digitized online using a 16-bit AD converter (National Instruments NI-6221, Austin, TX). Offline data extraction was completed with a custom-made MATLAB toolbox. A DS7A constant-current stimulator (Digitimer, Hertfordshire, UK) was used to deliver a mild electric stimulus. Sound was presented through headphones (Sennheiser HD 202, Wedemark, Germany). Pen and paper were used by participants to write down answers to one of the tasks included in the experiments (i.e., free recall task). The participant and researcher were the only ones present during data collection. The researcher was not blind to experimental conditions (within-subjects design) or to the study hypothesis.
Timing	Experiment 1: March - April 2019; Experiment 2: February - May 2021
Data exclusions	Experiment 1: 1 participant was excluded for not following instructions and 2 because of technical malfunction (e.g., problematic SCR electrodes that did not correctly register responses). We had preregistered the following exclusion criteria: "Participants experiencing technical malfunctions will be excluded, as will those that do not follow the instructions or do not complete the full task." Experiment 2: 2 participants were excluded due to technical malfunctions with the psychophysiological equipment and 21 participants were excluded for SCR non-responding (SCR amplitudes < 0.02µS on 75% of all trials), an exclusion criterion that we preregistered for the second experiment only (in addition to the criteria described for Experiment 1). However, in the manuscript we include analyses with

	both samples; one analysis including all participants and a second analysis with the excluded participants removed.
Non-participation	No participants dropped out/ declined participation.
Randomization	Participants were not allocated to experimental groups; within-subjects design.

Reporting for specific materials, systems and methods

We require information from authors about some types of materials, experimental systems and methods used in many studies. Here, indicate whether each material, system or method listed is relevant to your study. If you are not sure if a list item applies to your research, read the appropriate section before selecting a response.

Materials & experimental systems

n/a	Involved in the study
☒	☐ Antibodies
☒	☐ Eukaryotic cell lines
☒	☐ Palaeontology and archaeology
☒	☐ Animals and other organisms
☒	☐ Clinical data
☒	☐ Dual use research of concern
☒	☐ Plants

Methods

n/a	Involved in the study
☒	☐ ChIP-seq
☒	☐ Flow cytometry
☒	☐ MRI-based neuroimaging
